# Primary immunodeficiency

**DOI:** 10.1186/1710-1492-7-S1-S11

**Published:** 2011-11-10

**Authors:** Christine McCusker, Richard Warrington

**Affiliations:** 1 McGill University, Montreal, Quebec, Canada; 2 University of Manitoba, Winnipeg, Manitoba, Canada

## Abstract

Primary immunodeficiency disorder (PID) refers to a heterogeneous group of over 130 disorders that result from defects in immune system development and/or function. PIDs are broadly classified as disorders of adaptive immunity (i.e., T-cell, B-cell or combined immunodeficiencies) or of innate immunity (e.g., phagocyte and complement disorders). Although the clinical manifestations of PIDs are highly variable, most disorders involve at least an increased susceptibility to infection. Early diagnosis and treatment are imperative for preventing significant disease-associated morbidity and, therefore, consultation with a clinical immunologist is essential. PIDs should be suspected in patients with: recurrent sinus or ear infections or pneumonias within a 1 year period; failure to thrive; poor response to prolonged use of antibiotics; persistent thrush or skin abscesses; or a family history of PID. Patients with multiple autoimmune diseases should also be evaluated. Diagnostic testing often involves lymphocyte proliferation assays, flow cytometry, measurement of serum immunoglobulin (Ig) levels, assessment of serum specific antibody titers in response to vaccine antigens, neutrophil function assays, stimulation assays for cytokine responses, and complement studies. The treatment of PIDs is complex and generally requires both supportive and definitive strategies. Ig replacement therapy is the mainstay of therapy for B-cell disorders, and is also an important supportive treatment for many patients with combined immunodeficiency disorders. The heterogeneous group of disorders involving the T-cell arm of the adaptive system, such as severe combined immunodeficiency (SCID), require immune reconstitution as soon as possible. The treatment of innate immunodeficiency disorders varies depending on the type of defect, but may involve antifungal and antibiotic prophylaxis, cytokine replacement, vaccinations and bone marrow transplantation. This article provides a detailed overview of the major categories of PIDs and strategies for the appropriate diagnosis and management of these rare disorders.

## Introduction

Primary immunodeficiency disorder (PID) refers to a heterogeneous group of disorders characterized by poor or absent function in one or more components of the immune system. Over 130 different disorders have been identified to date, with new disorders continually being recognized [1,2]. Most PIDs result from inherited defects in immune system development and/or function; however, acquired forms have also been described [3]. It is important to note that PIDs are distinct from secondary immunodeficiencies that may result from other causes, such as viral or bacterial infections, malnutrition, or treatment with drugs that induce immunosuppression.

With the exception of immunoglobulin A (IgA) deficiency, PIDs are rare; the estimated prevalence of these disorders in the United States is approximately 1 in 1200 live births. IgA deficiency is the most common PID, occurring in approximately 1 in 300 to 1 in 500 persons [4].

The clinical presentation of PIDs is highly variable; however, most disorders involve increased susceptibility to infection. In fact, many PIDs present as “routine” infections (often of the sinuses, ears and lungs) and, therefore, may go undetected in the primary-care setting. The accurate and timely diagnosis of these disorders requires a high index of suspicion and specialized testing. Therefore, consultation with a clinical immunologist who is experienced in the evaluation and management of immunodeficiencies is essential, since early diagnosis and treatment are critical for preventing significant disease-associated morbidity and improving patient outcomes [5-7]. This article provides an overview of the major categories of PIDs as well as strategies for the timely identification, diagnosis and management of these disorders.

## Classification

PIDs are broadly classified according to the component of the immune system that is primarily disrupted: adaptive or innate immunity (see *An Introduction to Immunology and Immunopathology* in this supplement for more information on adaptive and innate immunity). Table 1 presents a select list of PIDs grouped according to this system [5,8].

**Table 1:** Classification of PIDs: examples and typical clinical presentations [5,8]

Classification and examples	Clinical presentation
**Disorders of adaptive immunity**	

**T-cell (cellular) immunodeficiency** ► IFN-γ/IL-12 ► AIRE mutations	Atypical mycobacterial and salmonella infections Mucocutaneous candidiasis (thrush) and autoimmune endocrinopathy

**B-cell (antibody-mediated) immunodeficiency** ► XLA ► CVID ► Selective IgA deficiency ► Specific antibody deficiency ► IgG subclass deficiency	Recurrent sinopulmonary infections with encapsulated bacteria Autoimmune disease and increased risk of malignancy in CVID

**CID** ► Wiskott-Aldrich syndrome	Thrombocytopenia with bleeding and bruising; eczema; recurrent bacterial and viral infections; autoimmune disease
► Ataxia telangiectasia	Chronic sinopulmonary disease; cerebellar ataxia (difficulty with control of movement); small, dilated blood vessels of the eyes and skin; malignancy
► DiGeorge syndrome	Hypoparathyroidism; seizures; cardiac abnormalities; abnormal facies; infection
► SCID • T^-^ , B^+^ – **γ**c deficiency – JAK3 deficiency • T^-^ , B^-^ – ADA deficiency – RAG 1/2 deficiency	Severe, recurrent opportunistic infections; failure to thrive; diarrhea; rash

**Disorders of innate immunity**	

**Phagocyte defects** ► Chronic granulomatous disease ► Hyper IgE syndrome ► Leukocyte adhesion deficiency	Severe infection; abscesses with granuloma formation Chronic dermatitis; recurrent, severe lung infections; skin infections; bone fragility; failure to shed primary teeth Recurrent, severe bacterial infections; poor wound healing; delayed separation of the umbilical cord

**Complement defects** ► Deficiency in early complement pathway components (C1q, C1r, C2, C4)	SLE–like syndrome, rheumatoid disease, multiple autoimmune diseases, infections
► Deficiency in late complement pathway components (C5, C6, C7, C8, C9)	Neisserial infections, SLE-like syndrome
► C3 and regulatory components	Recurrent infections with encapsulated bacteria

AIRE, autoimmune regulator; CVID, common variable immunodeficiency; IgG, immunoglobulin G; IgE, immunoglobulin E; IgA, immunoglobulin A; IFNγ, interferon-gamma; IL, interleukin; CID, combined immunodeficiency; SCID, severe combined immunodeficiency; XLA, X-linked agammaglobulinemia; SLE: systemic lupus erythematosus; JAK3, Janus kinase 3; ADA, adenosine deaminase; RAG, recombination activating gene

### Disorders of adaptive immunity

T cells and B cells are the primary cells of the adaptive immune system. B cells mediate antibody production and, therefore, play a major role in antibody-mediated (humoral) immunity. T cells, on the other hand, govern cell-mediated immune responses. Defects occurring at any stage of T-cell development, differentiation and maturation lead to T-cell (cellular) immunodeficiency disorders, while defects relating to B-cell development and/or maturation result in B-cell (antibody-deficiency) disorders. Since B-cell-mediated antibody production requires intact T-cell function, most T-cell defects lead to combined (B- and T-cell) immunodeficiency disorders (CIDs) [3,5].

### Disorders of innate immunity

Innate immune responses represent the first line of defense against potentially invading organisms. Appropriate recognition of threats and induction of the inflammatory cascade are essential steps in the removal of these organisms from the system. Failure of the innate system to identify pathogens delays the induction of the immune response and may worsen outcomes of infection.

Numerous cells and proteins are involved in the innate immune response including phagocytes (neutrophils and macrophages), dendritic cells, and complement proteins. Phagocytes are primarily responsible for phagocytosis, the process by which cells engulf and eliminate invading pathogens. Complement proteins function to identify and opsonize (coat) foreign antigens, rendering them susceptible to phagocytosis. Defects in the development and function of any of these elements of innate immunity may lead to PIDs.

## Clinical presentation

### T-cell and combined immunodeficiencies

The clinical manifestations of T-cell (cellular) disorders and CIDs will vary depending on the specific underlying defect in the adaptive immune response. Therefore, clinical suspicion is important for timely diagnosis of these disorders. Patients with specific T-cell defects may be lymphopenic (i.e., have abnormally low levels of lymphocytes) and neutropenic (i.e., have abnormally low levels of neutrophils). In the most severe forms of CID (also known as severe combined immunodeficiency [SCID]), there is a virtual lack of functional T cells and immune function. These disorders are rare and are generally categorized into whether there is an absence of T cells, but presence of B cells (T^–^ , B^+^), or an absence of both T and B cells (T^–^ , B^–^) (see Table 1). Natural killer (NK) cell numbers are also informative for determining the genetic phenotype of SCID [3,5]. However, normal T-cell numbers do not exclude the possibility of T-cell defects, and in patients with clinical presentations consistent with immunodeficiency, further investigations of T-cell function are warranted.

Patients with SCID usually present within the first year of life with chronic diarrhea and failure to thrive; severe, recurrent infections with opportunistic pathogens (e.g., *Candida albicans* [thrush], *Pneumocystis jiroveci*, or cytomegalovirus); and skin rashes. Some patients may also have associated neurological defects. SCID is a pediatric emergency since infection often leads to death and bone marrow transplantation (BMT) can be curative [3,5].

Other, less severe CIDs that do not characteristically lead to early mortality include Wiskott-Aldrich syndrome, DiGeorge syndrome, ataxia-telangiectasia, and X-linked lymphoproliferative disease. Patients with these disorders often present later in childhood with recurrent infections and clinical findings that vary depending on the specific syndrome (see Table 1). Autoimmunity and immune dysregulation are also frequent complications associated with these CIDs [3,5].

### B-cell immunodeficiencies

B-cell (antibody-deficiency) disorders are the most common type of immunodeficiencies, accounting for approximately 50% of all PID diagnoses [5]. They comprise a heterogeneous group of disorders characterized by an increased susceptibility to respiratory tract infections with bacteria, particularly *Streptococcus pneumoniae* and *Haemophilus influenzae.*

Patients usually present after 6 months of age with recurrent, and often severe, sinopulmonary infections such as otitis media, sinusitis, and pneumonia. Diarrhea, fatigue, autoimmune manifestations (particularly autoimmune cytopenias), and sensorineural hearing loss are also common [6,9]. Patients with humoral deficiency often have reduced or absent serum immunoglobulin levels, but may also show normal or increased serum immunoglobulin levels with abnormal function.

More than 20 antibody-deficiency disorders have been defined to date, however, many remain undefined. Typical disorders that fall into this category include: X-linked agammaglobulinemia (XLA; also known as Bruton’s agammaglobulinemia), common variable immunodeficiency (CVID), and selective IgA deficiency. XLA results from a mutation in the Bruton’s tyrosine kinase (Btk) gene, which is responsible for mediating B-cell development and maturation. The disorder is characterized by markedly reduced levels of circulating B-cells and serum IgG, IgA and IgM. Affected males usually present within the first 2 years of life with recurrent sinopulmonary infections and absent lymph nodes and tonsils [5,9]. CVID is a heterogeneous disorder characterized by markedly reduced serum concentrations of IgG, low levels of IgA and/or IgM, and poor or absent responses to immunization. The disorder affects males and females equally, and usually has a later age of onset than other antibody-deficiency disorders (i.e., > 10 years of age). It is associated with recurrent sinopulmonary infections, autoimmune and granulomatous disease, gastrointestinal complications and an enhanced risk of malignancy (e.g., lymphoma and gastric carcinoma). Some patients may also present with bronchiectasis (irreversible widening of portions of the bronchi resulting from damage to the airway wall), which is a common cause of morbidity and mortality in these patients [5].

Milder antibody-deficiency disorders, such as selective IgA deficiency, are associated with variably low serum levels of an immunoglobulin class or subclass and, in some cases, impairments in specific antibody formation. IgA deficiency, for example, is characterized by low or absent levels of serum IgA in the presence of normal levels of IgG and IgM. Only about one-third of these patients are particularly prone to infection [5].

### Innate immunodeficiencies

Patients with innate immunodeficiency disorders may present at any age, often with unusual or difficult to eradicate infections. The typical signs and symptoms of phagocyte disorders are severe pyogenic (puss-like) bacterial and fungal infections of the skin, respiratory tract, and internal organs, as well as painful sores around the mouth. Chronic granulomatous disease (CGD) is a common phagocyte defect. Hyper-IgE syndrome is another phagocyte disorder characterized by Staphylococcal infections of the skin, bone, and lungs, bony abnormalities and high IgE levels. (see Table 1) [3,5,8]. It has recently been found to result from a mutation in signal transducer and activator of transcription 3 (STAT3) which affects phagocytic cell recognition of *Staphylococcus* as well as osteoclast function involved in bone remodelling [10].

Of all the PIDs, complement deficiencies account for less than 1% of identified cases. Patients with these disorders tend to present with systemic autoimmune disease that resembles lupus erythematosus or with severe or recurrent infections with encapsulated organisms (see Table 1) [3,5,8].

## Diagnosis

**Figure 1:**
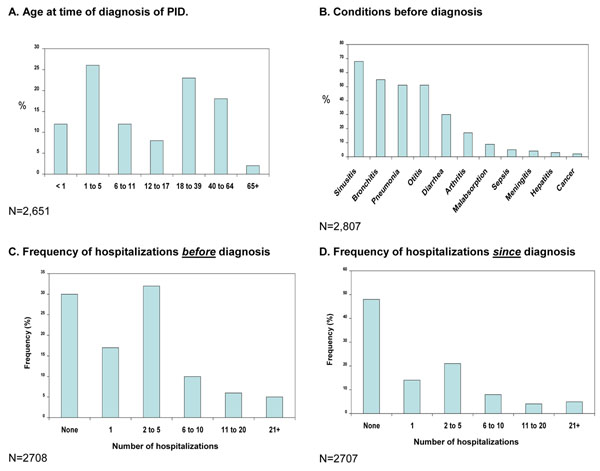
Results from the Immune Deficiency Foundation (IDF) national survey of PIDs [11]

As mentioned previously, early diagnosis of PID is critical for preventing significant disease-associated morbidity, and even mortality. However, a national survey of PID in the United States found that more than 40% of patients with these disorders were not diagnosed until adulthood (Figure 1A), despite the fact that many reported serious or chronic health conditions prior to diagnosis, such as sinusitis, bronchitis, and pneumonia (see Figure 1B) [11]. The importance of prompt recognition and management of PIDs is further highlighted by the rate of hospitalizations pre- and post diagnosis. Although 70% reported being hospitalized prior to diagnosis (Figure 1C), nearly half (48%) reported no hospitalization since diagnosis (Figure 1D).

A diagnosis of PID should be suspected in both children and adults who have recurrent pneumonias and/or ear, sinus and cutaneous infections as listed in Table 2. Although this Table does not provide a comprehensive list of all signs and symptoms of PID, patients meeting any of these criteria should be referred immediately to a clinical immunologist for further evaluation [7,12]. The immunologist will perform a comprehensive immune evaluation that often begins with a complete blood count (CBC) and blood smear. These tests are used to evaluate for the presence of lymphopenia, abnormal or unusual lymphocytes or phagocytic cells, and any associated gross hematologic abnormalities that may be indicative of PIDs. Significant lymphopenia, for example, may be the first indication of T-cell (cellular) immunodeficiency. Other important diagnostic tools include lymphocyte proliferation assays and flow cytometry which allow for the enumeration of B-cells, T-cells, and NK, and the evaluation of lymphocyte markers, T-cell variability, and adhesion receptors that may be associated with specific immune defects. Standard flow cytometry analysis is abnormal in most cases of SCID and in many cases of CID [6,13].

**Table 2:** The Jeffrey Modell Foundations' 10 warning signs of immune deficiency. [**12**]

1. ≥ 8 new ear infections with in 1 year. 2. ≥ 2 serious sinus infections within 1 year. 3. ≥ 2 months on antibiotics with little effect. 4. ≥ 2 pneumonias with in 1 year. 5. Failure of an infant to gain weight or grow normally. 6. Recurrent, deep skin or organ abscesses. 7. Persistent thrush in mouth or elsewhere on skin, after age 1. 8. Need for intravenous antibiotics to clear infections. 9. ≥ 2 deep-seated infections. 10. A family history of PID.

The initial evaluation of patients with suspected B-cell (antibody-deficiency) disorders involves the measurement of serum IgG, IgA, IgM, and IgE levels (note that the measurement of IgD is not useful for the diagnosis of PIDs). Serum levels that are clearly below age-appropriate reference values may be indicative of B-cell immunodeficiencies. However, some patients with these disorders have normal or only modestly reduced immunoglobulin levels; therefore, the best approach for confirming a diagnosis of an antibody-deficiency disorder is the measurement of serum specific antibody titers (usually IgG) in response to vaccine antigens. This approach involves immunizing a patient with protein antigens (e.g., tetanus toxoid) and polysaccharide antigens (e.g., pneumococcus) and assessing pre- and post-immunization antibody levels. In many PIDs, antibody responses to these antigens are diminished or even absent [6].

Neutrophil function assays (e.g., dihydrorhodamine response [DHR]) and stimulation assays for cytokine responses are helpful for confirming a diagnosis of innate disorders. For example, abnormal neutrophil oxidase function is usually indicative of CGD. Complement studies, which examine the level and/or function of specific complement proteins, are essential for the diagnosis of complement deficiency disorders. These studies should be performed by accredited laboratories that have demonstrated competence in these assays and experience in performing investigations into PID [5,6].

In some cases, more advanced testing involving specialized molecular methods may be required to confirm a diagnosis of PID [13]. Once the diagnosis is established, it is important that therapy be initiated as soon as possible, since delays can lead to permanent organ damage or even death from overwhelming infection [5].

## Treatment

The treatment of PIDs is complex and generally involves both supportive and definitive strategies (see Table 3). As such, therapy should be coordinated by an immunologist with expertise in the management of these disorders [5,7].

**Table 3:** Strategies for the treatment and management of PIDs.

	Supportive	Definitive
**CIDs/SCID**	► Ig replacement therapy (IV or SC) ► Antibiotic prophylaxis ► Antifungal prophylaxis ► Aggressive management of established infections ► Infectious precautions when hospitalized ► Withhold all live vaccines	► BMT ► HSCT ► Gene therapy a possibility for some SCIDs

**B-cell disorders**	► Ig replacement therapy (IV or SC) ► Antibiotic prophylaxis ► Antifungal prophylaxis depending upon etiology ► Hearing assessment ► Assessment of pulmonary status and function ► Close monitoring for co-morbidities	► Gene therapy is a potential future treatment in some patients

**Innate disorders**	► Antibiotic prophylaxis ► Antifungal prophylaxis ► Cytokine replacement (IFNγ) for CGD ► Vaccinations (e.g., meningococcal) ► Ig replacement is sometimes indicated	► BMT, e.g., for CGD ► Gene therapy is a potential future treatment

Ig, immunoglobulin; IV: intravenous; SC, subcutaneous; CID, combined immunodeficiency; SCID, severe combined immunodeficiency; IFNγ, interferon-gamma; BMT, bone marrow transplantation; CGD, chronic granulomatous disease; HSCT, hematopoietic stem cell transplantation

### SCID/CID

Initial therapy for patients with SCID or other CIDs is supportive and involves aggressive management of the established infection, immunoglobulin (Ig) replacement therapy (discussed in more detail in the next section), and antibiotic and antifungal prophylaxis to reduce the frequency and severity of infections. There is currently no standardized approach to the use of prophylactic antibiotics in patients with established PIDs since randomized, controlled studies in this area are lacking. Commonly used regimens are derived from studies focusing on the prevention of otitis media in children and include: sulfisoxazole**,** amoxicillin, trimethoprim-sulfamethoxazole (TMP-SMX) and azithromycin [3,5]. Patients with SCID should also be protected from exposure to infectious agents. In the hospital setting, protective isolation in positive pressure rooms is recommended. Furthermore, live attenuated vaccines (e.g., such measles/mumps/rubella/varicella, bacillus Calmette-Guerin, infant rotavirus, and oral polio virus) are contraindicated in patients with SCID as they can lead to severe, disseminated and fatal infections [5]. There is no risk of disseminated infections from killed or inactivated vaccines and, therefore, these may be administered according to routine indications and schedules in patients with PIDs [5].

Since SCID is fatal unless the underlying defect is corrected, definitive therapy with BMT or hematopoietic stem cell transplantation **(**HSCT**)** should be initiated as quickly as possible. When performed from a human leukocyte antigen (HLA)-identical sibling, these procedures lead to excellent long-term survival and long-lasting immune reconstitution. Good results have also been obtained with HLA-mismatched related donors when the procedures are performed within the first 3.5 months of life; however, less satisfactory outcomes have been noted in older patients [3,5]. Gene therapy, which involves introducing a functional copy of the patient's defective gene into appropriate cells, has also been shown to lead to immune reconstitution and improved survival in patients with certain SCIDs, such as adenosine deaminase (ADA) deficiency and SCID-X1 (an X-linked inherited SCID characterized by an early block in T-cell differentiation) [14]. Enzyme replacement therapy with weekly intramuscular injections of pegylated bovine ADA is also available for the management of patients with ADA deficiency [3].

### B-cell immunodeficiencies

The mainstay of therapy for most B-cell (antibody-deficiency) disorders is intravenous (IV) or subcutaneous Ig replacement therapy; in fact, many patients will require this treatment indefinitely. There are currently five Ig replacement products approved for the treatment of PID in Canada (see Table 4). IV and subcutaneous formulations are considered equally effective in reducing the frequency and severity of infections, and there is insufficient evidence to suggest that one product is superior to another [3,7]. When deciding on a specific product, patient preference should be taken into consideration. Some patients may prefer a subcutaneous formulation since therapy can be administered at home. Note that intramuscular Ig replacement therapy is not considered to be as effective as IV or subcutaneous therapy and, therefore, is not recommended for the treatment of PID [7].

**Table 4:** Ig replacement therapies for PID approved in Canada [**7**].

	Gammagard S/D, (Baxter)	Gamunex/IGIVnex, Talecris (Biotherapeutics)	Gammagard Liquid, (Baxter)	Privigen CSL, (Behring Canada)	Vivaglobin CSL, (Behring Canada)
**Formulation**	Lyophilized	Liquid	Liquid	Liquid	Liquid

**Administration**	IV	IV	IV	IV	SC

**Concentration**	5% or 10% upon reconstitution	9%-11%	9%-11%	10%	16%

**Shelf-life**	Not specified	36 months	36 months	Not specified	24 months

**Storage requirements**	Up to 25°C Do not freeze	2°C-8°C (36 mo), up to 25°C (6 mo) Do not freeze	2°C-8°C (36 mo), up to 25°C (for a single period of up to 9 mo within the first 24 mo from date of manufacture) Do not freeze	Room temperature (up to 25°C) Do not freeze Keep in the original carton to protect it from light	2-8°C (24 mo) Do not freeze Keep vials in storage box until use Do not administer if vial has been opened more than 4 h

**Infusion rate and dosage**	For 5% solution: 4 mL/kg/h, maximum (3.3 mg/kg/min, maximum) For 10% solution: 8 mL/kg/h, maximum (calculated rate:13.3 mg/kg/min, maximum)	0.14 mL/kg/min (14 mg/kg/min, maximum)	8 mL/kg/h maximum (calculated rate: 13.3 mg/kg/min, maximum)	0.08 mL/kg/ min, maximum (8 mg/kg/min)	100-200 mg/kg weekly; maximum volume of 15 mL per injection site at a rate of 20 mL/h Previous IV Ig dose ×1.37, then divide into weekly dose based on previous IV Ig interval

IV: intravenous; SC: subcutaneous

The recommended starting dose of Ig replacement therapy is 400–600 mg/kg/4 weeks for the IV formulation and 100–150 mg/kg/week for the subcutaneous formulation [7]. The most common adverse events associated with this therapy are headache, flushing, chills, myalgia, wheezing, tachycardia, lower back pain, nausea, and hypotension. In patients experiencing multiple adverse reactions to one product, consideration may be given to switching to another product or route of administration [7].

For patients with recurrent infections, prophylactic antibiotic therapy (particularly with agents that provide coverage of *Streptococcus pneumoniae* and *Haemophilus influenzae*) may also be needed in addition to Ig replacement therapy. Depending on the etiology of the specific B-cell disorder, prophylactic antifungal therapy may also be required. Since B-cell immunodeficiencies are often associated with sensorineural hearing loss and pulmonary complications, regular hearing assessments and monitoring of pulmonary status and function is recommended. As with primary T-cell defects, vigilance for malignancies and autoimmune disorders is also important in patients with B-cell disorders.

At present, there are no definitive management strategies that can be routinely recommended for patients with B-cell disorders. However, gene therapy is currently being investigated for some antibody deficiencies and may represent a future treatment option for these patients [14].

### Innate disorders

The management of innate disorders depends on the type of defect. For phagocyte disorders, therapy is primarily supportive and includes both antibiotic and antifungal prophylaxis. Cytokine replacement (e.g., interferon-gamma) and BMT have also been shown to be effective in patients with CGD. Gene therapy may also be a potential definitive treatment option in the future [5,14].

There is no specific definitive therapy for complement deficiencies. Treatment of these disorders focuses on antibiotic prophylaxis for the prevention of recurrent infections. Since some patients with complement disorders are at increased risk of meningococcal infections with *Neisseria meningitidis*, multivalent meningococcal vaccinations should also be considered. Pneumococcal and *Haemophilus influenzae* vaccines may also be needed in patients with frequent infections caused by encapsulated organisms.

## Prognosis

The prognosis of patients with PIDs varies depending on the etiology of the disorder. However, patient outcomes and long-term survival have improved significantly since the 1970s given our improved management of infections and early access to antibiotics, advances in BMT and HSCT techniques, and enhanced intensive care services. Furthermore, routine vaccinations provide herd immunity to those at risk, decreasing the circulation of infectious disease. Further progress in the diagnosis and management of PIDs is expected as research on the genes responsible for immunodeficiencies and the use of definitive treatments such as gene therapy continues.

## Conclusions

PID refers to a heterogeneous group of disorders that result from defects in immune system development and/or function. Although the signs and symptoms of PIDs are highly variable, most disorders involve increased susceptibility to infection, with many leading to significant disease-associated morbidity and mortality. Given the complexity of these disorders, referral to an immunologist is required for appropriate diagnosis and management. Severe disorders such as SCID requires definitive therapy for immune reconstitution (e.g., BMT, HSCT) as soon as possible. B-cell or antibody-deficiency disorders are the most common types of PIDs. The mainstay of treatment for patients with these disorders is Ig replacement therapy. Patients with innate immunodeficiency disorders often present with unusual or difficult to eradicate infections. Treatment varies depending on the type of defect (e.g., phagocyte disorder or complement deficiency), but may involve antifungal and antibiotic prophylaxis, cytokine replacement, vaccinations and BMT.

## Key take-home messages

► With the exception of IgA deficiency (prevalence = 1 in 300-500), PIDs are more frequent than previously believed, with an estimated prevalence of 1 in 1200.

► Clinical presentation is highly variable, but most disorders involve increased susceptibility to infection.

► PIDs should be suspected in patients with: recurrent sinus or ear infections or pneumonias within a 1 year period; failure to thrive; poor response to prolonged use of antibiotics; persistent thrush or skin abscesses; or a family history of PID.

► Consultation with a clinical immunologist is required to confirm the diagnosis of PID and to establish an appropriate treatment plan.

► SCID is fatal unless the underlying defect is corrected and, therefore, definitive therapy with BMT or HSCT should be initiated as quickly as possible.

► Ig replacement therapy is the mainstay of therapy for antibody-deficiency disorders, and is also an important supportive treatment for many patients with other forms of PID including SCID/CID.

► Antibiotic and antifungal prophylaxis are also recommended for many PIDs to prevent the frequency and severity of infections.

## Competing interests

Dr. Christine McCusker has been a scientific advisory board member for Baxter, CSL Behring and Talecris.

Dr Richard Warrington is the past president of the Canadian Society of Allergy & Clinical Immunology and Editor-in-Chief of *Allergy*, *Asthma & Clinical Immunology.* He has received consulting fees and honoraria from Nycomed, CSL Behring and Talecris.
